# Referral to Pain Specialists for Treatment of Intractable Mandibular Pain Caused by Osteonecrosis of the Jaw: A Case Series Report

**DOI:** 10.7759/cureus.26150

**Published:** 2022-06-21

**Authors:** Yujiro Hiraoka, Megumi Matsumura, Kaito Uryu, Takumi Sato, Junya Kusumoto, Masaya Akashi

**Affiliations:** 1 Oral and Maxillofacial Surgery, Kobe University Graduate School of Medicine, Kobe, JPN; 2 Oral and Maxillofacial Surgery, Kobe University Hospital, Kobe, JPN

**Keywords:** best supportive care, segmental mandibulectomy, intractable pain, mandibular nerve block, osteonecrosis of the jaw

## Abstract

Pain is a problematic symptom in patients with osteonecrosis of the jaws (ONJ). Effective pain management in patients with advanced ONJ still remains an unresolved issue. This case series report presents three patients who were referred to the pain clinic for treatment of intractable pain caused by ONJ. Two patients received mandibular nerve blocks and achieved pain relief. After referral to the pain clinic, these two patients underwent segmental mandibulectomy for ONJ. In the third patient, the effect of pain control was limited. Appropriate cooperation between the oral and maxillofacial surgeon and the pain specialist is essential for pain management in patients with advanced ONJ who experience intense pain.

## Introduction

Osteonecrosis of the jaws (ONJ) is a side-effect of medical treatment such as bone-modifying agents for bone metastasis of cancer or osteoporosis (medication-related osteonecrosis of the jaws (MRONJ)), or radiation therapy for head and neck malignancy (osteoradionecrosis of the jaws (ORNJ)). Both MRONJ and ORNJ cause serious symptoms such as trismus, chronic drainage, disfigurement, and severe pain [1]. These symptoms reduce patients’ quality of life. Pain is one of the most problematic symptoms in patients with ONJ [1-4]. One of the major goals of treatment for patients with ONJ is pain control [5].

Pain management is an increasingly recognized field because pain-related diseases are the leading cause of disability and disease burden globally [6]. The cooperation between oral and maxillofacial surgeons and pain specialists is important, particularly in older patients in whom surgery is difficult and who may also be treated with long-term administration of non-steroidal anti-inflammatory drugs (NSAIDs) because of comorbidities such as renal failure, especially in Japan’s super-aged society [7]. Therefore, this case series report presents three cases of patients who were referred to pain specialists for the treatment of intractable mandibular pain caused by ONJ, and discusses the perspective of appropriate pain management in patients experiencing severe pain caused by ONJ.

## Case presentation

**Figure 1:**
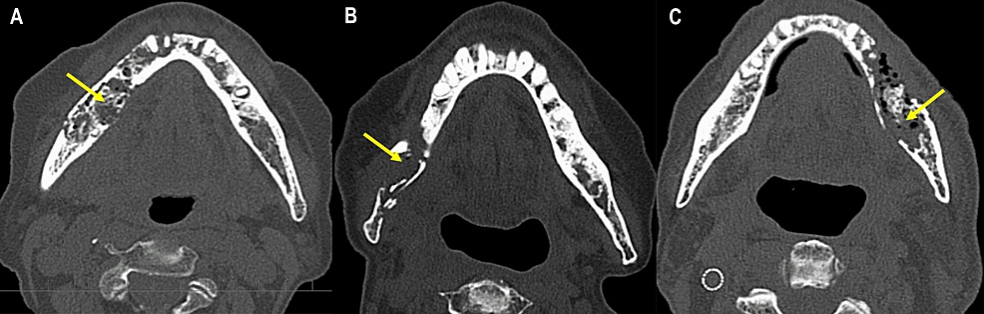
Axial computed tomographic images of Patient 1 (A), Patient 2 (B), and Patient 3 (C)

Three patients with intractable mandibular pain caused by ONJ were referred to the department of anesthesiology and pain clinic at our hospital from the department of oral and maxillofacial surgery between July 2016 and April 2021. Clinical characteristics are shown in Table 1. One patient was diagnosed with MRONJ (Patient 1) and two patients were diagnosed with ORNJ (Patient 2 and Patient 3). Patient 1 had severe kidney failure, and patients 2 and 3 had decreased renal function. Patients 1 and 3 were administered with anticoagulant. All three patients had experienced severe trismus before the referral to the pain clinic. Figure 1 shows severe bone destruction of the mandible in all three cases.

**Table 1:** Clinical characteristics of patients included in this study ONJ, osteonecrosis of the jaws; eGFR, estimated glomerular filtration rate; MRONJ, medication-related osteonecrosis of the jaws; ORNJ, osteonecrosis of the jaws; ^e ^This patient had no history of bone modifying agents

Patient	Sex	Age (years)	Type of ONJ	Primary disease	Comorbidity	eGFR	Anticoagulant use	Trismus
1	Female	72	MRONJ	Rheumatoid arthritis	Kidney failure, heart failure	16.9	Yes	Yes
2	Female	80	ORNJ	Pharyngeal melanoma	Coronary artery stenosis, osteoporosis^e^	56.7	No	Yes
3	Male	66	ORNJ	Oropharyngela squamous cell carcinoma	Liver cancer, esophageal cance,r cerebral infarction	100.1	Yes	Yes

Table 2 shows the treatments used for pain caused by ONJ. Drug therapy administered before referral to the pain clinic included acetaminophen, NSAIDs, anticonvulsants, and opioids. Treatments at the pain clinic were continuous or repeated mandibular nerve blocks combined with narcotic analgesics. After pain management by the pain specialists, Patient 1 and Patient 2 received surgery, while Patient 3 received the best supportive care.

**Table 2:** Treatments used for intractable pain and osteonecrosis of the jaw

Patient	Drug therapy before referral to pain clinic	Treatments by pain specialists Methods of mandibular nerve block/Type of narcotic analgesic	Treatments after referral to pain clinic
1	Acetaminophen, pregabalin	0.75 % ropivacaine injection twice daily for one week following continuous nerve block 0.25 % levobupivacaine/Fentanyl	Segmental mandibulectomy
2	Celecoxib, mirogabalin	1 % mepivacaine six times/Fentanyl	Segmental mandibulectomy
3	Acetaminophen, tramadol	1 % mepivacaine one time/Fentanyl	Best supportive care

Patient 1

A 72-year-old female had the right mandibular first molar extracted one year before attending our hospital. Three months after the extraction, she had severe pain in the right mandible and sensory reduction in the area served by the inferior alveolar nerve. She was diagnosed with MRONJ at the former hospital. Pain control by medication was difficult because the patient had severe kidney failure. After experiencing severe trismus, poor nutrition, and dehydration, the patient was admitted as an emergency hospitalization to our hospital. Panoramic x-ray and computed tomographic images showed severe bone destruction in the right mandible without pathological fracture (Figure 1A). The patient’s pain could not be controlled by the administration of oral analgesics and anticonvulsants (Table 1) or intravenous administration of antibiotics. One week after the emergency hospitalization, the patient was referred to the pain clinic at our hospital. A mandibular nerve block via a lateral extraoral approach was effective for pain at rest, but the severe pain reappeared. One week after the first mandibular nerve block, the pain specialist placed an indwelling catheter close to the foramen ovale under fluoroscopic guidance to provide a continuous mandibular nerve block. The detailed procedure has been previously described [8]. The continuous mandibular nerve block was effective for pain relief. The patient underwent surgery (segmental mandibulectomy and tracheostomy) about one month after hospitalization. Forty days after the surgery, she was discharged without pain or surgery-related complications.

Patient 2

An 80-year-old female received radiation therapy for a pharyngeal malignant melanoma eight years before her first visit to our department. She had ORNJ in the right molar region and underwent sequestrectomy via an intraoral approach under general anesthesia eight months after the first visit. After the sequestrectomy, her symptoms, including trismus, persisted. Her chronic pain gradually worsened. Seven months after the sequestrectomy, she was referred to the pain clinic for pain management. The patient received a mandibular nerve block weekly combined with narcotic analgesics. During pain management, a pathological fracture occurred (Figure 1B). Three months after the referral to the pain clinic, a segmental mandibulectomy was performed. The pain and infection disappeared, and the patient’s mouth opening increased. Her postoperative course was uneventful.

Patient 3

A 66-year-old male received radiation therapy for a right oropharyngeal squamous cell carcinoma and right neck lymph node metastasis eight years before his first visit to our department. He was diagnosed with severe ORNJ in the left mandible. He was followed up with conservative therapy because he had received chemotherapy for hepatocellular carcinoma. Although the mouth opening was only 15 mm during the follow-up, his pain was controlled by acetaminophen and tramadol. Eighteen months after the first visit, the patient was unable to ingest food orally because of the intense pain. Extensive bone destruction in the left mandible was apparent (Figure 1C). In the pain clinic, a mandibular nerve block was administered, and treatment with narcotic analgesics was started. The intractable pain was relieved, but the patient’s general condition rapidly deteriorated, exacerbated by malnutrition and uncontrolled hepatocellular carcinoma. The patient was then referred for best supportive care in another hospital.

## Discussion

One of the treatment goals for patients with ONJ is the control of pain. Surgery is not indicated for older patients or patients who have problematic comorbidities. Conservative pain management is extremely important. Two of the patients in this case series received continuous or repeated mandibular nerve blocks that succeeded in providing pain relief. Subsequently, both patients (1 and 2) were able to undergo radical extirpation of ONJ while their pain was controlled. In contrast, Patient 3 received a mandibular nerve block only once, because his prognosis was poor.

Mandibular nerve blocks are often performed for diagnostic, therapeutic, and anesthetic purposes for mandibular surgery [9]. Advantages include not only effective pain relief but also avoidance of the side effects associated with the use of opioids or NSAIDs [9]. Sawhney et al. [9] reported that an intermittent mandibular nerve block via the lateral extraoral approach was effective for pain relief after surgery for a mandibular parasymphyseal fracture. A continuous mandibular nerve block is effective for the relief of pain caused by MRONJ [8], mandibular fracture [10], and trigeminal neuralgia [11]. Dziadzko et al. [12] reported that a continuous bilateral nerve block was effective for painful trismus in patients with tetraplegia. Taken together, the referral to a pain specialist is reasonable not only for effective pain relief by nerve blocking but also to avoid excessive use of opioids and NSAIDs. However, it is noteworthy that patients with intense pain due to advanced ONJ often require extirpative surgery, aiming for complete control of pain and infection as seen in patients 1 and 2 in this report.

Patient 3 had been diagnosed with ORNJ well over a year previously. He could not receive extirpative surgery for ORNJ because he had received chemotherapy for hepatocellular carcinoma. The timing of the referral to the pain specialist may have been too late to effectively treat this patient’s severe pain. A recent systematic review suggests that specialist palliative case consultations in outpatients have a positive impact on pain control in cancer patients [13]. The European Society for Medical Oncology (ESMO) clinical practice guidelines mention that pain is common in cancer patients, particularly in the advanced stages, contributing to poor physical and emotional well-being [14]. One of the recommended invasive management techniques for refractory pain is the peripheral nerve block [14]. If pain is caused by a complication such as a pathological fracture (a common complication of advanced ONJ), a peripheral nerve block can be used. However, a peripheral nerve block as the principal pain treatment is rare, and pain management with systemic combined analgesia is usually necessary [14]. Oral and maxillofacial surgeons often monitor patients with advanced-stage ONJ who cannot undergo extirpative surgery. Therefore, appropriate cooperation is essential between oral and maxillofacial surgeons, pain specialists, and palliative care specialists in patients with intense pain and an inoperable status.

## Conclusions

Three patients were referred to the pain clinic, and received mandibular nerve blocks and narcotic analgesics. Two of the patients underwent surgery for ONJ followed by effective pain management, and one patient was referred to another hospital to receive best supportive care because of an advanced tumor. Appropriate cooperation between oral and maxillofacial surgeons and pain specialists is essential for pain management in patients with advanced ONJ.
